# New and Complete Manual of Auscultation and Percussion, Applied to the Diagnosis of Diseases

**Published:** 1836-04

**Authors:** 


### PART SECOND.

### Bibliographical Notices.

---

ART. I.—*Nouveau Manuel complet d'Auscultation et de Percussion, ou Application de l'Acoustique au Diagnostic des Maladies.* Par M. RACIBORSKI, M.D.P., Ex-chirurgien Militaire, Professeur de Médecine, Chevalier de la Croix Militaire d'Or de Pologne. *Paris*, 1835. 12mo. pp. 302.

*New and complete Manual of Auscultation and Percussion, applied to the Diagnosis of Diseases.* By M. A. RACIBORSKI, M.D., &c. Translated by WILLIAM FITZHERBERT, B.A. Cambridge. *London:* sm. 8vo. pp. 204. 1835.

THE writings of Williams, Stokes, Graves, Elliotson, Corrigan, and Hope, in this country, and of Colin, Louis, Andral, Bouillaud, Piorry, and others, on the continent, have perpetuated and familiarized the labours of the illustrious Laennec, so as to render any formal introduction of the subject-matter of the present work wholly unnecessary.

Auscultation and percussion, however, though familiar to the ears of British students and practitioners, are yet very far from having attained their acme of practical application, and we hail with pleasure every effort calculated to redirect attention to a study which we believe capable of removing much of the obscurity that too often attends the practice of the most enlightened members of a confessedly difficult profession.

As old advocates for the advantages of physical diagnosis, we will venture to commence the brief notice which we mean to give of M. Raciborski's work, by advising him, and all who write in defence of auscultation and percussion, not to weaken their arguments by seeking them in the extremes of ignorance and inattention, but rather to contrast the superiority of physical examination, with the practice of the most sagacious and enlightened, when deprived of such invaluable auxiliaries. All dogmatic assertions affecting questions foreign to those under discussion, and of admitted difficulty, should be carefully avoided; for we do not hesitate to say, that auscultation and percussion are *not* capable of "detecting the *least vestiges* of a pathological condition."

Percussion, it is well known, was discovered by Auenbrugger, but scarcely practised until revived by Corvisart. Its subsequent progress is mainly to be ascribed to the indefatigable and ingenious researches of Piorry, who not only demonstrated the superiority of mediate over immediate percussion, but successfully extended its utility to the examination of the abdominal as well as the thoracic organs. In England, we feel convinced that percussion has not been sufficiently attended to; both its difficulties and advantages have been much underrated, and we may almost say, that we have scarcely met a single practitioner who could practically avail himself of all the advantages it is capable of affording. Abdominal percussion is even now only occasionally practised, and we have often witnessed much uncertainty in the minds of practitioners whether a tumid abdomen was distended with air or fluid, whether the intestinal canal was loaded with solid or gaseous contents; whether tumours depended on a partially inflated intestine, or were the result of morbid deposit; doubts which could scarcely be agitated were percussion understood and properly applied. In the chapter on Percussion of the Thoracic Organs, the author has aimed at being minute, and no doubt intended to be clear; but he has fallen into the error of obscuring a simple subject by the multiplicity of details, and increased the confusion by pedantry of expression and superfluous periphrases. The uninitiated student will extricate with difficulty the valuable materials of the chapter from the ponderous verbiage which conveys them; and we regret that the difficulty has been rather increased than diminished by the translator. A small plate, with the outline of the different regions and subjacent organs, would have benefited the reader far more than pages of descriptive details.

Surely the portion of the chest corresponding to the lungs might be expressed more briefly and clearly, than as the "supra-diaphragmatic portion, splanchnological thorax, or thoracic cavity."

The necessity of attending to individual varieties is judiciously insisted upon. Our standard of what is healthy or morbid must be founded rather upon the transition in the sounds as we pass from organ to organ, than by comparison with any fundamental type, which indeed does not exist.

It might have been mentioned as a distinguishing mark of dulness of percussion arising from the liver or the lung anteriorly and inferiorly on the right side, that, if the patient draws a deep breath, the hue of dulness is depressed when it depends on the liver: if on the lung, it remains fixed. No allusion is made to the valuable information to be derived from ocular inspection of the chest; nor is the subject of mensuration of the thorax adverted to throughout the work: yet these two methods, although not strictly included under the terms which form the title of the volume before us, are almost inseparable from the practice of auscultation and percussion. To the former of these (inspection) we would particularly direct the attention of the student, as affording signs, at once the simplest, most easily learnt, and most certain. In the earlier stages of phthisis, it is often possible to detect a marked difference in the expansion of a part or the whole of one side of the chest when the stethoscope gives us little or no information, and this even (at least in females) without any removal of the clothes.

Much unnecessary importance is attached to the theory of M. Beau, who thinks that the sound of normal respiration is produced by the resonance of the whole column of air inspired or expired against the soft palate and adjoining parts. Authors, especially of Manuals, should recollect that there are certain hypotheses which may be safely left to refute themselves.

In enumerating the normal varieties of the respiratory murmur, the important modifications produced by age are entirely overlooked.

Puerile respiration in the adult is attributed by M. Raciborski to increased dilatation of the air-cells, which, if we may depend on the experiments of Cruveilhier, are only partially inflated during ordinary inspiration. The same observer has remarked that the cells of the upper lobe were most habitually dilated; an opinion advanced also by M. Broussais, who saw in this supposed local increase of function the cause of the frequent inflammation of this portion of the lung, and its peculiar liability to tubercles. The pathological and physiological part of these opinions is, we believe, in opposition to the best analysed facts, but it is not our province at present to refute them. As to the cause of the puerile respiration, it no doubt often depends on the physical conditions of other portions of the lung; but when it is general, and ceases and returns without any evident modification of the thoracic or diaphragmatic movements, it must probably be referred to increased functional activity, connected with some peculiar condition of the nervous system.

The author has omitted to notice what we believe to be highly important, in a practical point of view, to the young auscultator; viz. the necessity of paying attention to the *manner* of the patients' breathing. Many mistakes in diagnosis have, we are sure, arisen from the observer not being aware of the modifications in the respiratory murmur depending on the mode of inspiration and expiration. Care should be taken that no unusual effort be made; that the ribs are distinctly elevated and depressed; that the feeling of agitation frequently felt at a first examination has subsided; and that no unnecessary sounds are produced by the nose or mouth. The most contradictory results may be obtained by different observers who are unequally impressed with the importance of the above precautions, the same chest being to one an example of normal respiration, while to the other there is an almost total absence of respiratory murmur.

We also regret that no notice is taken of the *expiration* as a stethoscopic sign of value and importance. In the natural conditions of the lung, there are some slight variations as to intensity; but to the ear the expiration appears always deeply seated, shorter in duration, and more bronchial in its character, than the inspiration, and evidently produced in the larger bronchi. In proportion as the density of the pulmonary parenchyma is increased, the expiration becomes more and more superficial, approximates both in duration and character to the inspiration, of which it seems rather a repetition than an echo; and, in cases of emphysema and bronchitis of the smaller tubes, is often more distinctly heard than the inspiration. The fact of expiration being increased under the opposite conditions of condensation and rarefaction of the pulmonary structure, may be reconciled by the consideration, that increased expiration depends on diminished elasticity as well as increased density of the lung. The former condition renders the entrance of air into the imperfectly collapsed lung too gradual for the production of sound, while, during expiration, the superficies of the organ being compressed by the collapsing parietes, the contained air is more or less suddenly expelled through the smaller bronchi, and sound is the consequence. When the lung is condensed, the superior conducting power of the medium sufficiently explains the superficial state of the expiration. We purposely direct the reader's attention to the subject, satisfied that the study of the expiration is of peculiar value in relation to the detection of tubercular deposition in the substance of the lungs, before it is capable of being discovered either by percussion or modified inspiration.

M. Raciborski attributes emphysema to the mechanical action of the cough, distending the bronchi and air-cells, and destroying their elasticity. Under these circumstances, a portion of the inspired air remains imprisoned, while the fresh supplies are no longer capable of producing the murmur arising from the gradual vesicular expansion. We think this explanation in many instances correct, but cannot apply the term "Hypothetical" to the opinion of Laennec, who attributed emphysema to the rarefaction of the structure of the lung; for there are certainly cases in which the cells are not only distended, but extensively ruptured, in the way he has so accurately described.

The author's assertion that "the 'rubbing sound' (*bruit de frottement,*) is heard whenever a false membrane exists between the two pleuræ, whether it adheres to only one of them, or extends from one to the other," is far too absolute. In cases where adhesions are very recent and soft, where they are chronic and universal, where they are thinly scattered and very cellular in character, and in many other circumstances which interfere with the expansion of the affected side, auscultation is incapable of detecting their presence. It has been doubted whether the sound which has been aptly compared to the creaking of new leather, and which depends on a fibro-cartilaginous membrane, can be heard in the clavicular portions of the chest, when the movements of the lung are so limited. We can amply confirm the affirmative experience of Dr. Stokes on this subject; and can state that it not unfrequently accompanies the other signs of tubercular deposit; and, where it forms the parietes of a large excavation, and, consequently, where motion is impossible, it may be produced at pleasure by pressure on the corresponding portions of the chest, either by the ear or stethoscope.

The sibilant and sonorous bronchi are too hastily noticed. The young auscultator should, at least, be informed that the former are seated in the smaller bronchial tubes, the sonorous in the larger. It is justly remarked, that they are more distinctly heard after expiration than inspiration. Dance considered this to depend on the air being forced out more gradually, on account of the diminished energy of expiration; but we should rather say that the obstruction being seated in the bronchial tubes, these were acted upon during inspiration by the column of air filling their caliber and accumulating beyond them; while, during expiration, the distended vesicular portion of the lung being compressed by the collapsing parietes of the chest, the air is forced through the narrowed channels of the bronchi more rapidly than it had entered, and with a proportionate increase of sound. In emphysema, where a similar phenomenon is observed, it is owing to diminished elasticity rather than bronchial obstruction. Our author again cites the researches of M. Dance in explanation of the metallic tinkling. This accurate observer attributes it to the bursting of an air-bubble on the surface of liquid. That this is frequently the case is rendered probable by the numerous experiments of M. Beau, who ingeniously explains the fact of the sound being heard during expiration, coughing, talking, &c., in the following manner. In the majority of cases the pulmonary parenchyma surrounding excavations is hepatized and does not collapse during expiration; the air, expelled from the rest of the lung, rushes through the bronchi communicating with the cavern as well as with the trachea, and again acts locally on the former as in the act of inspiration. This may occasionally be the case, but it is equally certain that neither the existence of liquid effusion or bronchial communication are essential for the production of metallic tinkling; for, as Dr. Williams justly instances, it may be heard distinctly in the meatus auditorius externus, on covering the ear with the palm and striking the back of the hand with the finger; also in many other circumstances it is quite unconnected with the conditions alluded to by M. Beau. It is, in fact, the result of vibrations in a limited and more or less elastic excavation, closed or only slightly communicating with the external air.

No allusion is made to the amphoric resonance, an evident variety of the metallic tinkling, but requiring free communication with the exterior. We have heard it remarkably distinct in a tubercular excavation occupying the upper third of the left lung.

The phenomena of œgophony and pectoriloquy are hastily and imperfectly treated. M. R. ascribes the former to the compression exercised by a small quantity of fluid causing a closer application of the pleuræ to the parietes of the vesicles, forming a membrane more or less tense applied to the extremities of the respiratory passages. We shall not decide on the value of this at least doubtful hypothesis, but remark that œgophony has been distinctly heard when no fluid existed. It is also occasionally heard where the quantity of fluid is considerable, and under similar circumstances M. Piorry has remarked a peculiar ringing tone in the patient's supra-laryngœal voice. We have ourselves seen more than one example of this.

The fifth chapter is devoted to auscultation of the heart, and contains a valuable summary both of the structure and movements of that complicated organ. As it is almost literally extracted from the valuable work of M. Bouillaud, already so fully analysed in the present number; we shall here only notice a few points, which have been hardly touched on in that article.

In the discussions by M. Bouillaud on the subject of the bellows-sound, no notice is taken of the effect of an enlarged heart, acting on the pulmonary substance, in giving rise to this sound in the cardiac region. The sound occasionally produced by this cause has been supposed by experienced stethoscopists to arise from valvular disease, and is so similar in tone and *rhythm*, that without other distinctive characters the error is almost inevitable. The fact of a bellows sound occasionally being produced by the influence of the heart upon the lung, has been adverted to by Laennec, Law, Hope, and others, but it has been attributed to the compression of the thin layer of the lung intervening between the pericardium and thoracic parietes, and considered to be constantly synchronous with the respiration.

We are satisfied that the seat of compression is one or more of the larger bronchi, and that, when considerable, the impediment to the entrance of air into corresponding portions of the lung is sufficient to produce a succession of interrupted rushings of that fluid during the effort of respiration, which are not to be distinguished as sounds, from those depending on the heart itself.

When aware of the possibility of such a result, its reality may easily be determined by making the patient hold his breath, when the sound immediately disappears. Its existence on the right side or the left, its affecting the upper or lower lobes of one or both lungs, may assist our conclusions as to which side of the heart is principally affected. Interrupted respiration, voice and cough, are necessarily propagated in the lobe where the ramifications of the compressed bronchi terminate, and diminished respiration with accumulated bronchial secretion are among some of its secondary effects.

Though certainly difficult, we cannot coincide with the opinion of M. Raciborski, derived from M. Bouillaud, that all distinction between healthy sounds of the right and left sides of the heart is impossible. Their exact limits cannot certainly be defined, but the first sound is clearer under the sternum and a little to the right of that bone than over the region corresponding to the left ventricle. In cases of disease, as for instance hypertrophy of the left or dilation of the right ventricles, the distinction is much more palpable.

We are also desirous of impressing the young auscultator with the necessity of attending to the direction and extent of the cardiac sounds. When the lungs are healthy, the sounds from the right and left sides of the heart have their distinct mode of transmission through the chest, and attention to this is of great importance when unravelling the often difficult problem of which is the affected side. The relative intensity of the heart's action under the sternum, below the right and left clavicles, in the epigastrium, at the lower and posterior portions of the chest, should be carefully and successively studied, and if we conjoin the results with the comparative examination of the arterial and venous circulation, not omitting the important aid of percussion, we shall be able to form a far more accurate diagnosis of the nature and seat of cardiac disease than is generally thought attainable.

The same element of the direction of the sound will greatly assist us in our attempt to solve the very difficult problem as to which of the orifices of the heart is the seat of disease, in the case of anormal sounds.

Should the obstruction be situated in the sigmoid valves of the aorta, the anormal sound will be conveyed along the large arterial trunks, and be heard in the carotids and subclavians, while over the heart it will be limited to the region corresponding to the left ventricle; should, however, the auriculo-ventricular orifices be diseased, no sound will be transmitted through the vascular system, but it will be more diffused over the base of the heart, and in cases of dilated auricle will extend upwards towards the clavicle, at a certain distance from which it will suddenly cease. These remarks are principally applicable to the left side; the comparative rarity of valvular disease in the right diminishing the importance of correct diagnosis. We are quite aware that these distinctions are not without difficulty, and that they require time and attention to be appreciated; but we are equally aware of their practical utility, and without regard to minutiæ, we seriously abridge the advantageous application of auscultation and percussion.

Another novel application of auscultation has been discovered by Dr. Ficher, an American physician, who cites several cases of meningeal inflammation, where, by applying the ear to the head, a distinct "encephalic bellows-sound" was heard, depending, he believes, on the compression of the numerous arteries at the base of the brain. What strongly confirms the justice of this opinion is, that the sound is considerably diminished, or wholly disappears, when the circulation in the carotids is impeded or arrested. We have ourselves, however, no practical acquaintance with this fact.

A separate chapter is devoted to the important and interesting application of auscultation to pregnancy, and contains an intelligent summary of the principal results, though without reference to the ingenious researches of Dr. Kennedy. The cause of the placental sound has given rise to much diversity of opinion; and, since it is still *sub judice*, we shall detain the reader for a short time, while we endeavour to arrive at the most probable conclusion on the subject.

M. Kergaradec considered it produced by the passage of the blood through the placental vessels; Laennec placed it in the uterine arteries; M. Paul Dubois in the vascular system of the tissue of the uterus generally; Dr. Kennedy in the uterine arteries, aided probably by the circulation in the maternal placenta; and a very recent writer, in the enlarged uterine vessels corresponding to the portion immediately connected with the placenta.

Admitting any one of these hypotheses, perhaps the majority, if not all of the phenomena might, to a certain extent, be accounted for; but, believing that it is always better to extend the applications of known principles than to invent new ones, we shall give the preference to the opinion of M. Bouillaud, who attributes the placental murmur to the compression of one or more of the large vessels of the abdomen, as the hypogastric and external iliac arteries, by the uterus charged with the produce of conception. In the first place, it is not probable that the action of the mother's heart should be propagated so distinctly to the last ramifications of the uterine or placental vessels, and retain its intermittent character. Such a state of circulation would be different from any we are acquainted with in the tissue of our different organs, and difficult to reconcile with the fact of the fœtal circulation being wholly independent, as far as rhythm is concerned, of that of the mother. Under all other circumstances where a similar sound is produced, it may be traced either to compression or obstruction of the cavities of the heart or large vessels; and the nicest and most practised ear, as Dr. Montgomery observes, (Cyclopædia of Practical Medicine, art. *Pregnancy*,) cannot detect any difference between sounds produced by these conditions and those accompanying the pregnant uterus. This accurate observer has recorded a case of enlarged carcinomatous uterus, where the phenomenon was heard in the most perfect manner, and another instance of abdominal tumour compressing the aorta, where the sound was equally distinct; and he mentions the well-known fact, that it may at any time be imitated by pressing the end of the stethoscope over the region of the iliac vessels. M. Bouillaud has succeeded in displacing the placental murmur by making the patient lie alternately on the right or left sides; and we have ourselves distinctly produced the same effect. Our own experience is also opposed to the generally expressed opinion that the maximum point of intensity of the sound is always identical in the same individual; the fact of the sound being limited, in the great majority of instances, to the placental side of the uterus, would simply depend on only one artery being compressed; and probability is greatly in favour of that pressure coinciding with the heaviest portion of the womb.

A still more powerful argument in favour of the sound being the consequence of uterine pressure in one or both of the iliac arteries, is its being heard over the femoral artery, and on the side corresponding to the uterine murmur. By changing the position of the patient, we have been enabled distinctly to transfer the bellows-sound from one femoral artery to the other. It is, however, but right to mention that this result has been verified in only a single instance; no other opportunity for careful examination having presented itself since our attention was directed to this particular view of the subject.

The more diffused character of the sound, in the advanced stages of pregnancy, may be accounted for by the greater arterial surface exposed to the uterine pressure; and the sound being occasionally audible for a short time after the placenta is detached, but instantaneously ceasing when contraction has taken place, would in the first place depend on the volume of the uterus not having materially lessened, and in the second, on the conditions necessary for pressure no longer existing. We may also advert to the fact of the sound never being detected before the uterus has risen above the margins of the pelvis, or, in other words, until it would be capable of acting in the manner we have supposed; and every writer has described the placental murmur as most distinctly heard in one or both iliac regions. The changes in the character of the sound by the cessation of the fœtal circulation, by the removal of the placenta, the death of the fœtus, or tying the cord, may all be explained by the concomitant changes in the volume and weight of the uterus.

The fact that a bellows-sound can be detected in cases of bronchocele, as first noticed, we believe, by Dr. Forbes, is rather confirmatory than opposed to the views we are advocating. The carotid arteries are in precisely analogous situations to the iliac, and a bellows-sound existing or not existing under these circumstances, with all its various modifications, is simply depending on the interference or non-interference of the swelling with the large cervical arteries, and not upon any vascular peculiarity of the tumour itself. We have recently met with an instance in which the evidence of this was most satisfactory: the middle lobe being largely developed and presenting no bellows-sound, while the lateral lobes, of very moderate size, but passing between the sterno-cleido mastoidei muscles and the carotids, allowed it to be distinctly heard. In answer to the enquiry whether the same result will be obtained in active hypertrophy of other parts of the system, we would reply, that we are not aware of any bellows-sound having been heard where no arterial obstruction could be suspected. We feel therefore justified in suggesting the latter condition as the real source of the sound wherever it may be found.

The remainder of the work is devoted to the application of auscultation and percussion to the diagnosis of particular diseases.

The first chapter is devoted to diseases of the abdomen, the second to diseases of the chest.

The section on diseases of the liver is not sufficiently complete for every purpose of practical diagnosis. M. R. very properly insists on the advantages resulting from attention to the effects produced by change of position; but in addition to what we have already pointed out, as a distinguishing mark between dulness of sound arising from the liver or the hepatized right lung, he might have insisted upon the absence of bronchophony over dulness corresponding to the liver; and the state of the intercostal spaces, which are filled up and even prominent when acted upon by fluid, depressed when answering to solid hepatic enlargement. Dr. Stokes has also shown that where the liver is depressed below the edges of the ribs by pleuritic effusion, there is a distinct intermediate sulcus or depression formed by the imperfect adaptation of two convex surfaces, and which does not exist in cases of enlarged liver. The hypochondrium is also much less everted, the edges of the false ribs are less thrown outwards where the liver is simply depressed than where it is enlarged.

We have also derived assistance, particularly in stout subjects with enlargement of the left lobe, by defining the extent over which the cardiac sounds are propagated below the pulmonary thorax; finding them cease suddenly below the limits of the liver.

In addition to the author's remarks in the section on dilatation of the stomach, we would observe that where the volume of the organ is considerably increased, its limits may be ascertained by making the patient drink a large quantity of fluid, and comparing the results of percussion before and afterwards. Auscultation would also detect the situation of the descending fluid.

The author's observations on the application of percussion to the discovery of stercoraceous concretions, distention of the bladder and abdominal effusion, are well worthy the practitioner's attention.

The disappearance of the tympanitic sound of the cæcum, by change of position, is one of the most available and convenient tests for detecting inconsiderable peritoneal effusion. Auscultation has lately been applied to the diagnosis of peritonitis, the false membrane producing a rubbing sound similar to what occasionally exists in pleuritic affections. We have not yet verified this result, though analogy is in favour of its existence.

The section on bronchitis is clear and concise. M. Raciborski thinks, and we believe justly, that it would be difficult always to explain the productions of the sibilant rhonchus by the congestion and thickening of the mucous membrane; and that most generally, this rhonchus is occasioned by a thin lamina of viscid mucus lining the internal surface of the bronchi, the caliber of which is already more or less diminished by the effects of inflammation. The sudden appearance and disappearance of the sound after the expectoration of a small quantity of viscid mucus is thus easily explained and accounted for.

The physical conditions of the lung during the progress of pneumonia are well delineated. M. R. cites two observations to prove that crepitation is not invariably present in the earlier stages of the disease, and that bronchophony may appear and disappear without being either preceded or followed by this usually attending rhonchus. To account for this, M. R. supposes that there was considerable congestion of the vesicular parietes and their consecutive obliteration. In the examples of peripneumonia notha so well described by Piorry, both the crepitous rattle and bronchophony were absent. Allied to the above facts are the interesting observations of Dr. Hudson on typhoid pneumonia, in the Dublin Medical Journal, for July, 1835. The absence of crepitation and bronchophony is ascribed by Dr. H. to the compression of the air cells by sanguineous congestion, at once interfering with visceral expansion, and leaving a disadvantageous medium for transmission of sound. In these cases bronchophony, if subsequently present, would result from the consolidation of the effused fluid.

A still more remarkable deviation from the usual physical signs, is "a tympanitic clearness on percussion over a solidified lung, without air being present in the pleura." It varies, says Dr. H., in degree rather than in kind from what we hear when percussing the empty stomach or cæcum, and resembles the metallic tinkling occasionally found in a tubercular cavity; it is also at times as clear as in a case of pneumothorax. This physical paradox is said to have been verified by dissection in four instances, and the clearness and accuracy of Dr. H.'s details entitle the subject to attention. Does it depend, asks Dr. H., on the transmission of the gastric resonance through the lung; or on the facility with which the vibrations of the air in the bronchi are communicated to a lung *solid throughout*, and therefore not permitting the loss of such vibration? For ourselves we can only say, that if the fact is undeniable, which we may be permitted to doubt, it is equally inexplicable.

The enumeration of the physical signs of pleurisy are too limited in their range. The state of the intercostal spaces, the increased size of the chest, and the effect of effusion upon the heart, liver, and spleen, should be presented to the student's attention in conjunction with the signs furnished by auscultation and percussion.

In the section on tubercles the author has advanced what we believe to be very erroneous ideas of the etiology and progress of phthisis; and the diagnosis of tubercles by auscultation and percussion appears to us to be very incompletely laid down. Indeed, in all the earlier and more important periods of the disease, so desirable a result would appear impossible; for he remarks, that it is only when tubercles acquire a *considerable volume*, and form *large masses*, that percussion and auscultation can enable us to presume their presence. No reference is made to the important facts, that tubercles are almost invariably first deposited in the upper lobes; that when in a crude and miliary state, comparative and carefully executed percussion under the clavicle will reveal their presence; that auscultation is also capable of detecting coexisting variations of the respiratory murmur; and that even before percussion is sensibly affected, an attentive study of the expiration may clearly indicate a very inconsiderable amount of morbid deposition. We are aware that all this requires long practice and close attention fully to seize and appreciate; but how greatly is the value of signs enhanced when they point out an early and often remediable state of disease, over those which tell us our only task is to alleviate the passage to the grave! It is our accurate knowledge of the locality of pulmonary tubercles, which enables us to avail ourselves of the slight physical changes detected by auscultation and percussion, changes which would be comparatively negative, were tubercles equally developed in every portion of the lungs. Pectoriloquy, cavernous respiration, metallic tinkling, amphoric resonance, &c. are all, we freely admit, valuable additions to our stethoscopic phenomena, but they too frequently possess acoustic rather than pathological interest, and have very generally been studied to the exclusion of the less palpable but more valuable signs which attend the earlier stages of disease.

The concluding portions of the volume refer to the organs of circulation; and for the reasons already given we shall pass them over without any notice, observing merely that the observations on diseases of the heart are both hurried and incomplete.

The extent of our remarks on this small volume must be attributed to the importance of the subject. M. Raciborski's manual possesses few claims to originality. It is, however, an intelligent compilation of the labours of MM. Piorry and Bouillaud, and, as such, we are enabled to recommend it. It is certainly true that the author has failed to complete the picture he has himself drawn in his preface of what constitutes a good manual. In a future edition he will add to the reputation he has already acquired, by simplifying many of the details, excluding all unnecessary matter, and extending his remarks to every means of furthering physical examination. He has displayed much want of acquaintance with the labours of other than Parisian observers; his arrangement is diffuse, his description unequal, and his expressions are often pedantic; but he is still entitled to be considered as a successful promoter of the interests of auscultation and percussion.

To Mr. Fitzherbert the student is indebted for the English translation; and if being faithful and accurate constitute merit, he deserves oui praise. We regret, however, that the task was not intrusted to one more familiar with the subject, and more capable of amending and supplying the author's deficiencies. The translator appears to have forgotten that we have our own terms and technicalities, and that auscultation and percussion, though originating on the Continent, have long since found in England advocates and authors of no mean reputation, who have adopted a nomenclature rather more agreeable and useful to the English reader than one composed of words which he can neither understand or pronounce. Mr. Fitzherbert has evidently improved in his translation as he proceeded, but there are many passages the meaning of which is at best extremely obscure. Such defects must be remedied before the work can become popular in England. While the want of reference by the author to the writers of this country has not escaped remark, no effort has been made to supply the deficiency, nor is there a single criticism or addition of any value throughout the volume.

These remarks are not dictated by any inclination to find fault, or with the design of wounding the feelings of Mr. Fitzherbert, whose diligence and general accuracy we acknowledge, but to impress him and others with the conviction that a translation ought to be something more than a servile copy of the original, and that if reputation is sought for, it must be achieved by greater industry and exertion than what is necessary for accomplishing a mere interchange of words.

